# Letter to the editor: ‘Evaluation of the Bluetooth-enabled Scanbo device for point-of-care measurement of blood pressure and blood glucose: A cross-sectional pilot study in an urban slum of Bengaluru’

**DOI:** 10.1016/j.fhj.2025.100481

**Published:** 2025-11-06

**Authors:** Parth Aphale, Himanshu Shekhar, Shashank Dokania

**Affiliations:** Dr. D.Y. Patil Vidyapeeth (Deemed to be University), Pimpri, Pune, Maharashtra, India

To the Editor,

We read with great interest the article by Karthikeyan *et al* titled ‘Evaluation of the Bluetooth-enabled Scanbo device for point-of-care measurement of blood pressure and blood glucose: A cross-sectional pilot study in an urban slum of Bengaluru’ published in the *Future Healthcare Journal*.^1^ The authors are to be commended for conducting this timely and significant study, which provides valuable insights into the potential utility of novel point-of-care (POCT) devices in resource-limited settings. The findings of high diagnostic accuracy and reliability for the Scanbo device are particularly encouraging for improving non-communicable disease management in India.

However, as scholars in the field of clinical device validation, we would like to offer a few points for clarification that, if addressed, could further strengthen the evidence base for the Scanbo device. Firstly, the study’s methodology for blood pressure validation warrants some enquiry. While the Omron device is a clinically reliable tool, it is not considered the gold standard for device validation studies. International protocols from bodies like the AAMI and ESH typically mandate the use of a trained observer with a mercury sphygmomanometer as the reference standard.^2^ We are curious about the rationale for using an automated Omron device as the reference and how this might influence the concordance results. Could the authors clarify if the Omron device itself was re-validated against a gold standard for the purpose of this study?

Secondly, we noted a lack of specific detail regarding the ‘diagnostic lab method’ used for blood glucose measurement. The study mentions that venous blood samples were sent to a laboratory, but a comprehensive validation requires specifying the exact analytical method (eg, hexokinase/glucokinase or glucose oxidase) and the brand of the laboratory analyser used. This information is crucial for reproducibility and for confirming that the reference method is indeed a true gold standard.^3^ The absence of this detail makes it challenging to perform a robust critical appraisal of the blood glucose findings.

Lastly, we would like to seek clarification on the measurement protocol. The study states that blood pressure readings were taken consistently in the order of Omron followed by Scanbo. While a 1-min interval was maintained, this fixed order could potentially introduce a sequence bias, particularly a ‘white-coat’ effect or the patient’s reaction to the first measurement.^4^ A randomised cross-over design, where the order of device use is alternated, would have provided a more rigorous assessment and helped to mitigate this bias. Furthermore, we observed a potential inconsistency in the statistical analysis: while the introduction mentions a hypertension cutoff of 140 mmHg for SBP, and the corresponding ROC curve analysis appear to use a lower cutoff of 129 mmHg. A clarification on the specific clinical criteria used for the analysis would be appreciated.

In conclusion, this pilot study represents a crucial step toward validating a new generation of digital health tools for community-based care.^5^ Addressing these methodological points would not only enhance the transparency and academic rigor of the research but also instil greater confidence in the device’s clinical application.

## CRediT authorship contribution statement

**Parth Aphale:** Writing – review & editing, Writing – original draft, Supervision, Conceptualization. **Himanshu Shekhar:** Writing – review & editing, Writing – original draft, Visualization. **Shashank Dokania:** Writing – review & editing, Writing – original draft.

## Declaration of competing interest

The authors declare that they have no known competing financial interests or personal relationships that could have appeared to influence the work reported in this paper.
